# Beyond eosinophil counts: organ involvement patterns across hypereosinophilic syndrome subtypes in a single-center cohort

**DOI:** 10.3389/fimmu.2026.1933052

**Published:** 2026-08-19

**Authors:** Stefania Nicola, Simone Negrini, Luca Lo Sardo, Anna Quinternetto, Flavia Matys, Elena Gervasio, Monica Fornero, Iuliana Badiu, Giovanni Rolla, Luisa Brussino

**Affiliations:** 1 SCDU Immunologia e Allergologia, AO Ordine Mauriziano di Torino, Torino, Italy; 2 Dipartimento di Scienze Mediche, Università degli Studi di Torino, Torino, Italy

**Keywords:** cardiac involvement, diagnostic delay, eosinophil (EOS), eosinophilia, HES, hypereosinophilc syndrome, organ damage, two-tailed approach

## Abstract

**Introduction:**

Hypereosinophilic syndrome (HES) is defined by persistent hypereosinophilia Q5 associated with eosinophil-mediated organ damage. However, the relationship between HES etiology and organ-involvement patterns remains incompletely defined, particularly in non-hematological forms. This study aimed to describe organ involvement across HES subtypes and to explore clinical associations with multiorgan disease.

**Methods:**

In this retrospective single-center study, 105 patients referred for hypereosinophilia were classified after a standardized two-tailed diagnostic work-up as reactive HES, single-organ HES, lymphocytic-variant HES (L-HES), overlap HES, idiopathic HES, myeloid HES, or HEus. Demographic data, absolute eosinophil count (AEC) at onset, organ domains, organ burden, and exploratory associations with multiorgan disease were analyzed.

**Results:**

Mean age at onset was 54.6 ± 18.9 years, and 60.0% of patients were male. Thirteen patients were classified as HEus and had no detectable organ involvement, whereas all HES categories showed at least one involved organ domain. Among recorded organ domains, lung involvement was the most frequent (39.0%), followed by gastrointestinal (30.5%) and cutaneous involvement (29.5%). Peripheral nervous system involvement occurred in 19.0% of patients, whereas cardiac, renal, thrombotic, and central nervous system involvement occurred in 6.7%, 4.8%, 1.9%, and 1.0%, respectively. Multiorgan involvement occurred in 34 patients (32.4%).

**Discussion:**

Organ burden differed significantly across subtypes, with overlap HES, mainly driven by eosinophilic granulomatosis with polyangiitis (EGPA), showing the highest multiorgan burden. L-HES and idiopathic HES were predominantly associated with cutaneous disease, whereas single-organ HES involved only the gastrointestinal tract or lung. AEC correlated with the number of involved organs and was strongly associated with multiorgan involvement. Organ involvement in HES is common, heterogeneous, and unevenly distributed across subtypes. Integrating etiological classification with systematic organ phenotyping may improve risk stratification and guide diagnostic and follow-up strategies.

## Introduction

Hypereosinophilic syndrome (HES) is characterized by persistent blood and/or tissue hypereosinophilia associated with eosinophil-mediated organ damage. Over time, its definition has evolved from a diagnosis of exclusion requiring prolonged observation toward a clinically driven entity centered on the early recognition of tissue injury (1–3). Contemporary frameworks, including the 2024 SIAAIC statement, emphasize an integrated approach in which etiologic classification is not merely descriptive, but supports the identification of patients at risk of organ involvement and informs targeted diagnostic and therapeutic strategies (4).

A clinically relevant distinction is typically made between hematologic and non-hematologic HES. Hematologically characterized forms are generally supported by more established diagnostic pathways, including bone marrow assessment, cytogenetics, molecular testing, and lymphocyte immunophenotyping when appropriate. In contrast, non-hematologic HES comprises a heterogeneous spectrum of reactive, overlap, organ-restricted, and idiopathic conditions in which eosinophils often act as downstream effectors of diverse pathogenic processes. Beyond its classificatory value, this distinction may influence disease mechanisms, clinical presentation, and the pattern, timing, and detectability of organ involvement.

Organ damage represents the defining feature of HES and a major determinant of morbidity. Although the skin, lungs, gastrointestinal tract, heart, and nervous system are among the most frequently affected sites, virtually any organ may be involved. Importantly, eosinophil-mediated injury may be clinically silent or poorly symptomatic, particularly in its early stages. Cardiac, gastrointestinal, pulmonary, neurologic, renal, or vascular involvement may therefore remain under-recognized in the absence of targeted evaluation. Accordingly, the absence of symptoms does not reliably exclude organ damage, underscoring the need for systematic and comprehensive baseline phenotyping, as supported by the PHLEOS/INHES framework and by recent real-world data on the two-tailed diagnostic approach (5, 6).

Despite increasing recognition of the systemic burden of HES, the relationship between underlying etiology and organ tropism remains incompletely defined. Available evidence on organ involvement according to HES subtype is still limited and is largely driven by selected populations or hematologically characterized forms, whereas data across non-hematologic HES categories remain fragmented. This gap limits the ability to anticipate organ-specific risks, tailor diagnostic work-up, and optimize monitoring strategies in this heterogeneous population.

The present study aims to characterize patterns of organ involvement across HES and HEus subtypes in a single-center cohort, with a specific focus on non-hematologic HES categories, and to assess whether distinct etiologic categories are associated with specific organ profiles. The cohort was recruited in a tertiary Immunology and Allergy unit, a referral setting that helps explain the predominance of non-hematologic and organ-restricted phenotypes.

By addressing this underexplored area, we seek to refine the link between etiologic classification and clinically meaningful phenotyping, with potential implications for diagnostic accuracy, risk stratification, and longitudinal management.

## Patients and methods

### Study design and population

This retrospective, single-center observational study was conducted at the Advanced Unit of Immunology and Allergy, A.O. Ordine Mauriziano, Turin, Italy. Electronic medical records of patients referred for hypereosinophilia between January 2010 and December 2025 were reviewed.

Hypereosinophilia was defined as an absolute eosinophil count (AEC) >1,500 cells/µL documented on at least two separate occasions. Patients were eligible if they had completed a structured diagnostic work-up for suspected hypereosinophilic syndrome (HES) and, following completion of the diagnostic assessment, fulfilled criteria for HES or hypereosinophilia of undetermined significance (HEus) (5).

Exclusion criteria were: transient or non-sustained hypereosinophilia; incomplete diagnostic evaluation; lack of informed consent for the use of clinical data; and hypereosinophilia attributable to clearly defined transient or alternative conditions not requiring HES-oriented evaluation. Reactive HES was distinguished from transient secondary eosinophilia by persistence of hypereosinophilia, evidence of organ involvement, and the need for HES-oriented evaluation.

### Diagnostic work-up and HES classification

Diagnostic evaluation was reviewed and harmonized according to the previously described “two-tailed approach” (6), which integrates assessment of the underlying cause of hypereosinophilia with evaluation of eosinophil-related organ involvement. In patients evaluated before formal implementation of this framework, available clinical, laboratory, imaging, functional, and histological data were retrospectively reviewed to confirm that classification and organ-involvement assessment met the same predefined criteria. The diagnostic work-up included clinical assessment, laboratory testing, immunological and molecular investigations, imaging, functional studies, and histological evaluation when indicated. Core investigations included complete blood count with differential, serum biomarkers, autoimmune screening, and parasitological assessment where appropriate. Molecular testing for clonal eosinophilia was performed when indicated, together with targeted imaging and organ-specific investigations guided by clinical presentation or abnormal screening findings (6).

Patients were classified into the following categories according to integrated clinical, laboratory, molecular, imaging, functional, and histological findings: reactive HES, overlap HES, myeloid HES, lymphocytic-variant HES, idiopathic HES, single-organ eosinophilic HES, and HEus, in line with contemporary consensus definitions and structured HES frameworks (4, 5). In brief, myeloid HES was defined by evidence of clonal or molecular abnormalities consistent with a myeloid neoplasm; lymphocytic-variant HES by aberrant T-cell populations and/or clinical-laboratory features consistent with a lymphocytic variant; reactive HES by identifiable secondary causes; and overlap HES by features shared with defined eosinophilic or immune-mediated disorders. Idiopathic HES was diagnosed when no underlying cause was identified despite comprehensive evaluation. Potential diagnostic overlaps, including between idiopathic HES and early-stage EGPA, were resolved through integrated review of all available clinical, serological, imaging, histological, and longitudinal information. Patients were assigned to EGPA or another defined overlap disorder only when sufficient disease-specific features were present; otherwise, after exclusion of alternative causes, they remained classified as idiopathic HES.

HEus was defined by persistent hypereosinophilia in the absence of an identifiable cause and without evidence of eosinophil-related organ damage after completion of the diagnostic work-up (5).

### Data collection and assessment of organ involvement

For each patient, demographic data, relevant comorbidities, past medical history, clinical manifestations attributable to eosinophilic disease, HES subtype, laboratory parameters, AEC at diagnosis, and treatment history were collected. AEC at disease onset/diagnosis was defined as the value recorded at the first HES-oriented evaluation, before initiation of disease-specific therapy whenever available. AEC values were analyzed both in the overall cohort and according to HES subtype.

Organ involvement was defined as clinical, laboratory, radiological, functional, or histological evidence of tissue damage or organ dysfunction attributable to eosinophil-mediated inflammation, based on integrated clinical and diagnostic assessment. Briefly, pulmonary involvement was defined by compatible respiratory manifestations and/or abnormal imaging or functional findings attributable to eosinophilic disease; gastrointestinal involvement by compatible symptoms with endoscopic, histological, or specialist assessment support; cutaneous involvement by compatible skin manifestations, with biopsy confirmation when available; peripheral nervous system involvement by compatible neurological symptoms and/or electrophysiological evidence; cardiac involvement by compatible clinical, biomarker, electrocardiographic, echocardiographic, or cardiac imaging findings; renal involvement by compatible urinary, biochemical, imaging, or histological abnormalities; central nervous system involvement by compatible neurological manifestations and neuroimaging findings; and thrombotic involvement by objectively documented arterial or venous thrombosis considered related to eosinophilic disease (6).

Lung and cardiac involvement were systematically evaluated in all patients through first-level laboratory and instrumental assessments (6). Additional organ-specific investigations were performed when clinically indicated or prompted by abnormal screening findings, including dermatological evaluation with biopsy for cutaneous manifestations, upper and/or lower gastrointestinal endoscopy with histology, neurological assessment with electromyography/electroneurography or brain magnetic resonance imaging, and hematological evaluation with bone marrow biopsy when indicated (6).

Each organ domain was recorded as involved or not involved based on the available clinical and diagnostic assessment. The total number of involved organs was calculated for each patient, and multiorgan involvement was defined as involvement of two or more distinct organ systems. Organ involvement was analyzed both overall and after stratification by HES subtype.

### Statistical analysis

Continuous variables were summarized as mean ± standard deviation or median with interquartile range, as appropriate based on distribution assessed using the Shapiro–Wilk or Kolmogorov–Smirnov test. Categorical variables were expressed as absolute counts and percentages.

Comparisons between two groups were performed using Student’s t-test or the Mann–Whitney U test, as appropriate. Comparisons across HES subtypes were performed using one-way ANOVA or the Kruskal–Wallis test for continuous variables, and the chi-square test or Fisher’s exact test for categorical variables. No *post-hoc* pairwise comparisons were performed after the omnibus ANOVA or Kruskal–Wallis tests. Subtype-specific organ associations were examined separately in exploratory 2×2 analyses, with multiplicity addressed using the false discovery rate procedure described below.

Correlations between AEC and the number of involved organs were evaluated using Pearson’s or Spearman’s correlation coefficients, as appropriate. Exploratory analyses were conducted to assess associations between clinical variables and organ involvement, including multiorgan disease. Univariable logistic regression analyses were performed including HES subtype, AEC, demographic variables, and relevant clinical features.

In the presence of sparse 2×2 subtype-by-organ comparisons, including cases of zero cells or complete separation, odds ratios and 95% confidence intervals were estimated using the Haldane–Anscombe correction. For exploratory subtype-by-organ association analyses, p values were adjusted for multiple testing using the Benjamini–Hochberg false discovery rate procedure and reported as q values.

All tests were two-sided, and a p value <0.05 was considered statistically significant. Statistical analyses were performed using IBM SPSS Statistics for Windows, version 28.0.

### Ethics

The study was approved by the local ethics committee Comitato Etico Territoriale Interaziendale “A.O.U. Città della Salute e della Scienza di Torino” — protocol code CET00189/2026 — and was conducted in accordance with the Declaration of Helsinki and Good Clinical Practice guidelines. All patients provided informed consent for the use of their clinical data for research purposes.

## Results

### Study population and demographic characteristics

The study cohort comprised 105 patients evaluated for hypereosinophilia and classified after a standardized diagnostic work-up (**Table 1**). The mean age at disease onset was 54.6 ± 18.9 years, with a median age of 59 years [IQR 46–69]. Overall, 63 patients were male and 42 were female, corresponding to 60.0% and 40.0% of the cohort, respectively.

**Table 1:** Demographic characteristics and macro-category distribution by HES subtype.

Subtype	n (%)	Age at onset, mean ± SD	Age at onset, median [IQR]	Female, n (%)	Male, n (%)
Overall cohort	105 (100.0)	54.6 ± 18.9	59 [46–69]	42 (40.0)	63 (60.0)
Reactive HES	26 (24.8)	55.4 ± 17.6	58.5 [46.5–69.8]	6 (23.1)	20 (76.9)
Single-organ HES	24 (22.9)	43.8 ± 21.4	40 [25–67]	11 (45.8)	13 (54.2)
L-HES	16 (15.2)	64.1 ± 12.0	63 [53–72]	7 (43.8)	9 (56.2)
Overlap HES	15 (14.3)	58.9 ± 9.1	57 [53–64]	7 (46.7)	8 (53.3)
Heus	13 (12.4)	50.4 ± 21.7	60 [26–69]	4 (30.8)	9 (69.2)
Idiopathic HES	10 (9.5)	62.7 ± 22.0	75 [46–80]	7 (70.0)	3 (30.0)
Myeloid HES	1 (1.0)	46	46 [46–46]	0 (0.0)	1 (100.0)

Data are n (%) unless otherwise specified. HEus, hypereosinophilia of undetermined significance; HES, hypereosinophilic syndrome; L-HES, lymphocytic-variant HES.

Age and sex distributions varied across HES subtypes. Patients with lymphocytic-variant HES (L-HES) and idiopathic HES showed the highest mean ages at onset, 64.1 ± 12.0 and 62.7 ± 22.0 years, respectively, whereas patients with single-organ HES were younger, with a mean age of 43.8 ± 21.4 years. Patients with overlap HES had a mean age at onset of 58.9 ± 9.1 years, while those with HEus had a mean age of 50.4 ± 21.7 years. The single patient classified as having myeloid HES was 46 years old at onset.

Sex distribution also differed across subtypes. Male predominance was observed in reactive HES and HEus, where males accounted for 76.9% and 69.2% of cases, respectively. In contrast, idiopathic HES showed a female predominance, with women representing 70.0% of patients. Overlap HES showed a slight male predominance, with 8 males and 7 females.

### Distribution of HES subtypes

Macro-category frequencies are summarized in **Table 1**, whereas the detailed etiologic classification is reported separately in **Table 2** to distinguish broad HES categories from their underlying subtypes.

**Table 2:** Detailed etiologic classification of HES and HEus.

Detailed subtype	n (%)
Single-organ HES	24 (22.9)
L-HES	16 (15.2)
Parasite-related reactive HES	14 (13.3)
Overlap HES – EGPA	14 (13.3)
Heus	13 (12.4)
Idiopathic HES	10 (9.5)
Drug-induced reactive HES	5 (4.8)
Asthma-associated reactive HES	5 (4.8)
Other reactive HES	2 (1.9)
Overlap HES - IgG4-related disease	1 (1.0)
Myeloid HES	1 (1.0)

Data are n (%) unless otherwise specified. EGPA, eosinophilic granulomatosis with polyangiitis; HEus, hypereosinophilia of undetermined significance; HES, hypereosinophilic syndrome; L-HES, lymphocytic-variant HES.

Using the detailed classification, single-organ HES represented the largest individual group, accounting for 24 cases (22.9%), followed by L-HES in 16 cases (15.2%). Among reactive forms, parasite-related reactive HES was the most common subgroup, occurring in 14 patients (13.3%), followed by drug-induced reactive HES and asthma-associated reactive HES, each observed in 5 patients (4.8%), and other reactive HES in 2 patients (1.9%). Among overlap forms, eosinophilic granulomatosis with polyangiitis (EGPA) represented the majority, with 14 patients (13.3%), while IgG4-related disease was observed in 1 patient (1.0%).

### Absolute eosinophil count at disease onset

**Figure 1:**
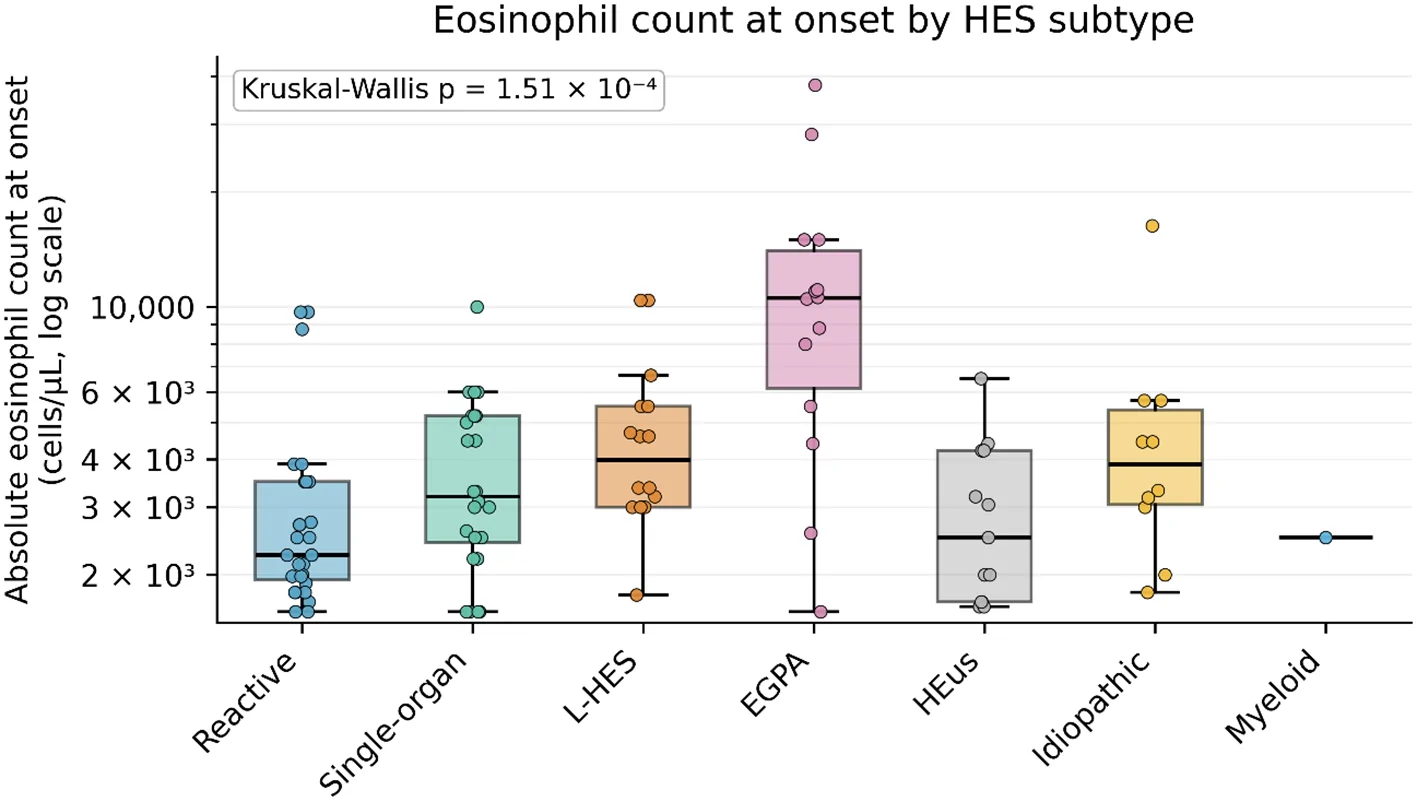
Absolute eosinophil count at onset by HES subtype. Boxes indicate the median and interquartile range; individual points represent patients. The y-axis is shown on a logarithmic scale. Single-patient subgroups (myeloid HES) are shown descriptively. AEC, absolute eosinophil count; EGPA, eosinophilic granulomatosis with polyangiitis; HES, hypereosinophilic syndrome; HEus, hypereosinophilia of undetermined significance; L-HES, lymphocytic-variant HES.

The median absolute eosinophil count (AEC) at diagnosis was 3,200 cells/µL [IQR 2,200–5,500], with a mean value of 4,911 ± 5,094 cells/µL and a range of 1,600–38,000 cells/µL. AEC differed significantly across subtypes, as assessed by the Kruskal–Wallis test (H = 26.90, p = 1.51 × 10^-4^) (**Figure 1**). The highest values were observed in patients with EGPA, who had a mean AEC of 12,162 ± 9,944 cells/µL and a median value of 10,550 cells/µL. In contrast, lower values were observed in HEus, with a mean AEC of 2,985 ± 1,488 cells/µL and a median value of 2,500 cells/µL. Given the presence of a single case, the cohort of IgG4-related disease was excluded and therefore not represented in **Figure 1**.

Intermediate AEC values were observed in the remaining subtypes. Patients with reactive HES had a mean AEC of 2,990 ± 2,095, while those with single-organ HES had a mean value of 3,819 ± 2,008 cells/µL. In L-HES, the mean AEC was 4,754 ± 2,532 cells/µL, with a median of 3,985 cells/µL. Patients with idiopathic HES had a mean AEC of 4,990 ± 4,199 cells/µL and a median of 3,885 cells/µL. The single patient with myeloid HES had an AEC of 2,500 cells/µL.

### Overall organ involvement

Organ involvement was documented in 92 of 105 patients (87.6%), whereas absence of organ damage was restricted to the HEus group, which included 13 patients (12.4%). Among the overall cohort, 58 patients (55.2%) had single-organ involvement and 34 patients (32.4%) had multiorgan involvement, defined as involvement of two or more organs. Specifically, 16 patients (15.2%) had two organs involved, 12 patients (11.4%) had three organs involved, and 6 patients (5.7%) had four organs involved.

Among recorded organ domains, the most frequently involved system was the lung, affected in 41 patients (39.0%). Gastrointestinal involvement was observed in 32 patients (30.5%), while skin involvement was documented in 31 patients (29.5%). Peripheral nervous system involvement occurred in 20 patients (19.0%). Less frequent manifestations included cardiac involvement in 7 patients (6.7%), renal involvement in 5 patients (4.8%), thrombotic involvement in 2 patients (1.9%), and central nervous system involvement in 1 patient (1.0%) (**Table 3**).

**Table 3:** Overall organ involvement.

Variable	n (%)
Skin involvement	31 (29.5)
Gastrointestinal involvement	32 (30.5)
Lung involvement	41 (39.0)
Peripheral nervous system involvement	20 (19.0)
Cardiac involvement	7 (6.7)
Renal involvement	5 (4.8)
Central nervous system involvement	1 (1.0)
Thrombotic involvement	2 (1.9)
Other/unspecified organ involvement	11 (10.5)

### Organ burden according to HES subtype

Organ burden differed significantly across HES subtypes, as assessed by the Kruskal–Wallis test (H = 65.84, p = 2.91 × 10^-^¹²) (**Table 4**). By definition, all HEus patients had no organ involvement. Among HES categories, overlap HES showed the highest organ burden, driven mainly by EGPA, with a mean of 2.67 involved organs and a median of 3 organs [IQR 2–3]. Multiorgan involvement was observed in 14 of 15 patients with overlap HES (93.3%). Within this group, 13 of 14 patients with EGPA had involvement of two or more organs.

**Table 4:** Organ burden across HES subtypes.

Subtype	n	Mean number of organs	Median [IQR]	Any organ, n (%)	≥2 organs, n (%)
Reactive HES	26	1.46	1 [1–2]	26 (100.0)	8 (30.8)
Single-organ HES	24	1.00	1 [1–1]	24 (100.0)	0 (0.0)
L-HES	16	1.94	2 [1–3]	16 (100.0)	8 (50.0)
Overlap HES	15	2.67	3 [2–3]	15 (100.0)	14 (93.3)
HEus	13	0.00	0 [0–0]	0 (0.0)	0 (0.0)
Idiopathic HES	10	1.60	1 [1–2]	10 (100.0)	4 (40.0)
Myeloid HES	1	1.00	1 [1–1]	1 (100.0)	0 (0.0)

L-HES also showed frequent multiorgan disease, with 8 of 16 patients (50.0%) having involvement of two or more organs. Multiorgan involvement was observed in 4 of 10 patients with idiopathic HES (40.0%) and in 8 of 26 patients with reactive HES (30.8%). By contrast, all patients with single-organ HES had involvement limited to one organ, and no cases of multiorgan disease were observed in this subgroup. The single patient with myeloid HES had one organ involved.

As expected based on classification criteria, absence of organ involvement was restricted to HEus, whereas all HES categories showed at least one involved organ domain.

### Organ-specific involvement according to HES subtype

**Figure 2:**
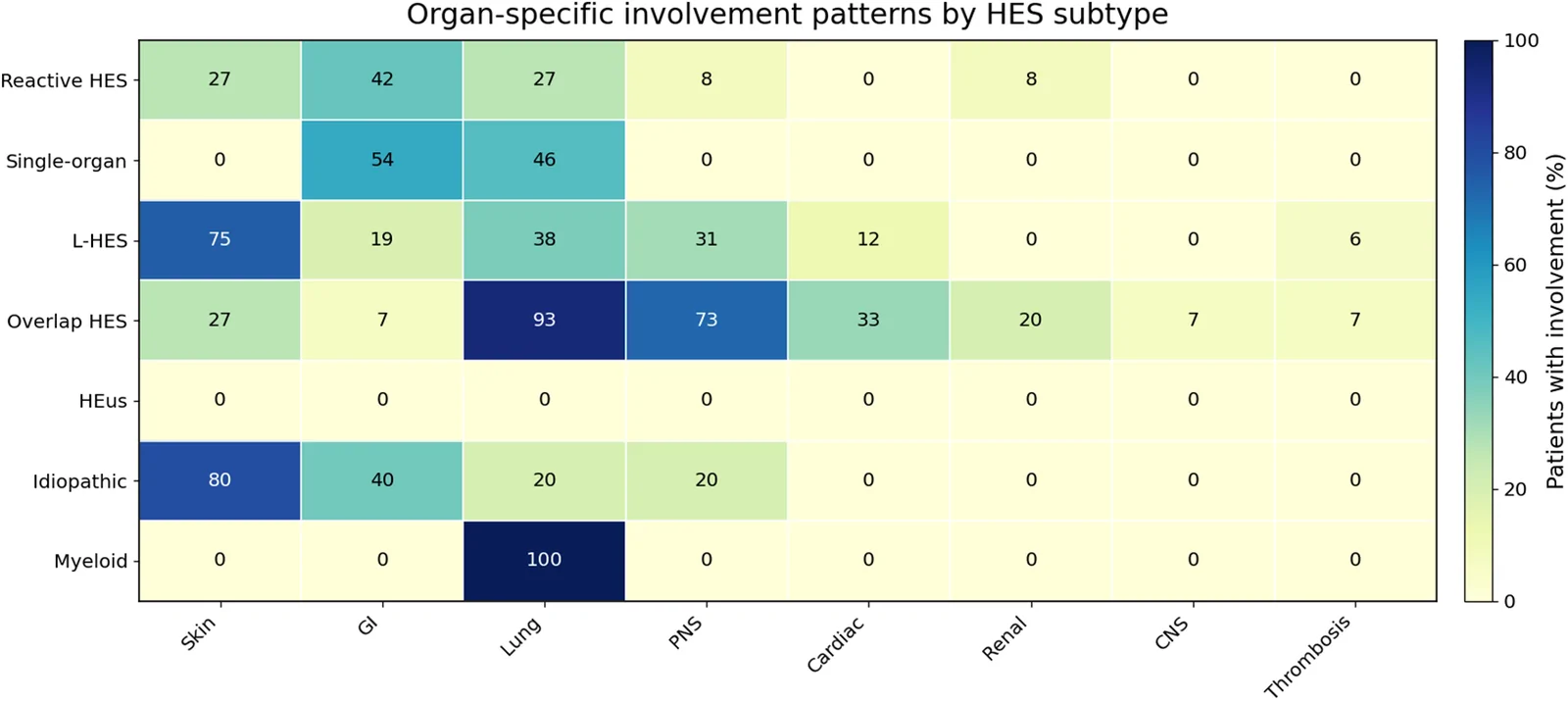
Organ-specific involvement patterns across HES subtypes. Cell values indicate the percentage of patients with involvement of each organ domain within each subtype. CNS, central nervous system; GI, gastrointestinal; PNS, peripheral nervous system.

Distinct organ-involvement patterns were observed across HES subtypes (**Figure 2**). In reactive HES, gastrointestinal involvement was the most common manifestation, occurring in 11 of 26 patients (42.3%). Skin and lung involvement were each observed in 7 patients (26.9%), while peripheral nervous system and renal involvement occurred in 2 patients each (7.7%). No cardiac, central nervous system, or thrombotic involvement was documented in this subgroup.

In single-organ HES, involvement was restricted to either the gastrointestinal tract or the lung. Gastrointestinal involvement was present in 13 of 24 patients (54.2%), whereas lung involvement was present in 11 patients (45.8%). No skin, neurological, cardiac, renal, central nervous system, or thrombotic involvement was recorded in this group.

Patients with L-HES showed a predominantly cutaneous phenotype. Skin involvement was documented in 12 of 16 patients (75.0%). Lung involvement occurred in 6 patients (37.5%), peripheral nervous system involvement in 5 patients (31.2%), gastrointestinal involvement in 3 patients (18.8%), cardiac involvement in 2 patients (12.5%), and thrombotic involvement in 1 patient (6.2%). No renal or central nervous system involvement was observed in L-HES.

In overlap HES, lung involvement was present in 14 of 15 patients (93.3%), driven by the EGPA subgroup, in which lung involvement was universal. All EGPA patients had asthma, and 6 patients (42.9%) also had transient parenchymal infiltrates. Peripheral nervous system involvement was observed in 11 patients, corresponding to 73.3% of overlap HES and 78.6% of EGPA patients. Cardiac involvement occurred in 5 patients (33.3%), skin involvement in 4 patients (26.7%), and renal involvement in 3 patients (20.0%). Gastrointestinal, central nervous system, and thrombotic involvement were documented in 1 patient each (6.7%).

By classification, HEus patients had no organ involvement in any organ domain. In idiopathic HES, skin involvement was the predominant manifestation, occurring in 8 of 10 patients (80.0%). Gastrointestinal involvement was observed in 4 patients (40.0%), lung involvement in 2 patients (20.0%), and peripheral nervous system involvement in 2 patients (20.0%). No cardiac, renal, central nervous system, or thrombotic involvement was observed in idiopathic HES. The single patient with myeloid HES had lung involvement only.

### Exploratory associations with organ involvement

**Figure 3:**
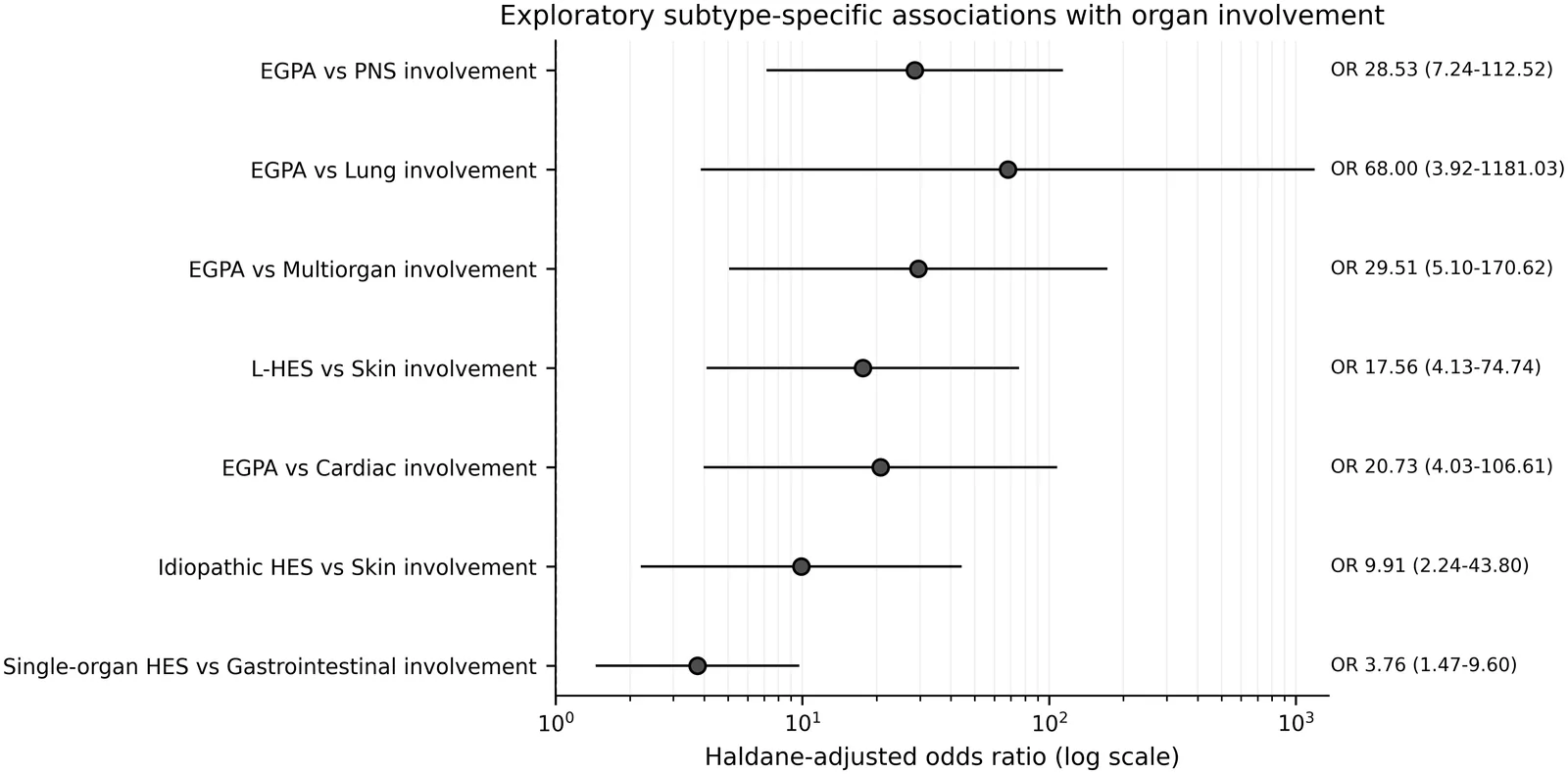
Exploratory subtype-specific associations with organ involvement. Points represent Haldane-adjusted odds ratios, and horizontal lines indicate 95% confidence intervals. The x-axis is shown on a logarithmic scale. CI, confidence interval; EGPA, eosinophilic granulomatosis with polyangiitis; HES, hypereosinophilic syndrome; L-HES, lymphocytic-variant HES; OR, odds ratio; PNS, peripheral nervous system.

Exploratory analyses identified associations between HES subtype, AEC, and organ involvement. **Figure 3** shows only the associations that remained significant after FDR correction. AEC at onset was positively correlated with the number of involved organs. Spearman correlation analysis showed a moderate positive association between AEC and organ burden (ρ = 0.431, p = 4.34 × 10^-6^).

In logistic regression analysis, log_10_-transformed AEC at onset was strongly associated with higher odds of multiorgan involvement, defined as involvement of two or more organs (OR 50.20, 95% CI 8.06–312.79, p = 2.73 × 10^-5^). Age at onset showed a weaker but statistically significant association with multiorgan involvement, with each additional year of age associated with a modest increase in the odds of multiorgan disease (OR 1.03, 95% CI 1.00–1.05, p = 0.034). Female sex was not significantly associated with multiorgan involvement (OR 1.29, 95% CI 0.56–2.95, p = 0.552).

Subtype-specific analyses showed strong associations between EGPA and several organ-involvement outcomes. EGPA was associated with peripheral nervous system involvement, which was present in 11 of 14 EGPA patients (Haldane-adjusted OR 28.53, 95% CI 7.24–112.52, p = 1.86 × 10^-7^; q = 7.80 × 10^-6^). EGPA was also associated with lung involvement, which occurred in all EGPA patients; because of complete separation, the Haldane-adjusted OR was 68.00 (95% CI 3.92–1181.03, p = 3.84 × 10^-7^; q = 8.07 × 10^-6^). EGPA was further associated with multiorgan involvement, observed in 13 of 14 patients (Haldane-adjusted OR 29.51, 95% CI 5.10–170.62, p = 7.33 × 10^-7^; q = 1.03 × 10^-5^), and with cardiac involvement, present in 5 of 14 patients (Haldane-adjusted OR 20.73, 95% CI 4.03–106.61, p = 3.72 × 10^-4^; q = 0.002).

L-HES and idiopathic HES were associated with skin involvement. In L-HES, skin involvement was documented in 12 of 16 patients (75.0%) and was associated with this subtype in exploratory analysis (Haldane-adjusted OR 17.56, 95% CI 4.13–74.74, p = 6.93 × 10^-6^; q = 7.28 × 10^-5^). In idiopathic HES, skin involvement occurred in 8 of 10 patients (80.0%) and was similarly associated with the subtype (Haldane-adjusted OR 9.91, 95% CI 2.24–43.80, p = 0.001; q = 0.006).

Finally, single-organ HES was restricted to gastrointestinal or pulmonary involvement.

Overall, these exploratory analyses indicated that organ involvement was unevenly distributed across HES subtypes. Overlap HES, driven mainly by EGPA, was characterized by prominent pulmonary, neurological, cardiac, and multiorgan involvement; L-HES and idiopathic HES were predominantly associated with skin involvement; single-organ HES mainly involved the gastrointestinal tract or lung; and HEus represented the subgroup without detectable organ damage.

## Discussion

In this real-world cohort of 105 patients with hypereosinophilia and hypereosinophilic syndromes, organ involvement was frequent, heterogeneous, and unevenly distributed across HES subtypes. The study population showed the expected demographic profile of an adult HES cohort, with a mean age at onset of approximately 55 years and a slight male predominance. The mean absolute eosinophil count (AEC) at disease onset was markedly elevated, albeit with wide interindividual variability, underscoring that peripheral eosinophilia alone does not fully capture disease burden. Consistent with classification criteria, patients classified as HEus had no detectable organ involvement, whereas all HES categories showed at least one involved organ domain. Therefore, the clinically informative findings of this study relate primarily to the distribution and burden of organ involvement across HES subtypes (1–4).

The distribution of HES subtypes in this cohort illustrates the complexity of current HES taxonomy in clinical practice. Reactive HES represented the largest etiologic group, followed by single-organ HES, lymphocytic-variant HES (L-HES), overlap forms, HEus, idiopathic HES, and a very small proportion of myeloid HES. This pattern likely reflects the recruitment setting and the inclusion of a broad spectrum of non-hematologic and organ-restricted phenotypes. Importantly, these findings support the view that HES classification should not be regarded as a purely etiologic exercise. Rather, etiologic classification and systematic organ phenotyping should be considered complementary and interdependent dimensions of the diagnostic process, as emphasized by consensus recommendations and the Phleos/INHES framework, which integrates both aspects in a structured manner (4, 5). Such a multidimensional approach is particularly relevant in a heterogeneous condition such as HES, where clinical manifestations and underlying mechanisms only partially overlap. This conservative classification strategy was particularly important for patients with possible early EGPA: cases without sufficient disease-specific features were retained as idiopathic HES rather than reclassified on the basis of eosinophilia or organ pattern alone.

Pulmonary involvement emerged as the most frequent manifestation in the overall cohort, followed by gastrointestinal and cutaneous involvement, with peripheral nervous system involvement also relatively common. In contrast, cardiac, renal, thrombotic, and central nervous system manifestations were less frequent. This distribution is consistent with prior real-world data indicating that the lung, skin, gastrointestinal tract, and peripheral nervous system represent common sites of disease expression in HES (6–9). Notably, patterns of organ involvement differed substantially across HES subtypes. Single-organ HES predominantly affected the gastrointestinal tract or lung; L-HES and idiopathic HES were mainly associated with cutaneous involvement; reactive HES showed a prominent gastrointestinal component; and eosinophilic granulomatosis with polyangiitis (EGPA) displayed the highest organ burden, with a distinct multiorgan phenotype involving the lung, peripheral nervous system, and heart, consistent with the multisystem nature of this condition (10). These findings suggest that patterns of organ involvement may provide clinically informative clues to the underlying HES subtype and help guide targeted diagnostic evaluation.

Another key finding was the association between eosinophil burden and organ involvement. AEC differed across HES subtypes, correlated with the number of involved organs, and was associated with multiorgan involvement in exploratory analyses. These findings support using AEC to calibrate diagnostic vigilance: higher counts should prompt a more intensive search for occult organ damage. However, AEC cannot replace direct organ phenotyping, because clinically relevant involvement occurred even at comparatively lower counts, whereas persistent hypereosinophilia may remain organ-silent in HEus. AEC should therefore be regarded as a risk marker that informs the urgency and breadth of investigation, not as a stand-alone measure of tissue injury (6, 11).

These findings have direct clinical implications. Patients presenting with elevated AEC should undergo systematic evaluation for organ involvement, even in the absence of specific symptoms. This is particularly relevant for organs such as the lung, peripheral nervous system, and heart, where early disease may be clinically silent yet associated with substantial long-term morbidity. Conversely, the absence of symptoms should not be considered sufficient to exclude organ involvement or HES in patients with persistent hypereosinophilia. Longitudinal follow-up may also be warranted in HEus, as the absence of detectable organ damage at baseline does not necessarily preclude subsequent evolution toward overt HES. Although therapeutic considerations were beyond the primary scope of this study, accurate identification of organ involvement remains clinically relevant in light of available targeted therapies, including anti–IL-5 therapy, which has demonstrated efficacy in selected HES populations (12, 13). Overall, these data support a two-dimensional diagnostic strategy in which etiologic classification and organ assessment are integrated from the earliest stages of evaluation (4–6, 14).

The very low prevalence of myeloid HES in this cohort warrants consideration. Only one patient was classified within this subtype, a finding that likely reflects referral patterns and cohort composition rather than the true epidemiology of clonal eosinophilic disorders. Indeed, myeloid HES is more commonly identified in hematology-focused settings with systematic molecular testing, whereas immunology- or internal medicine-based cohorts tend to capture a broader spectrum of reactive, lymphocytic, idiopathic, overlap, and organ-restricted forms (7, 8, 15). This observation underscores the impact of referral bias and highlights the importance of multidisciplinary collaboration to ensure comprehensive diagnostic evaluation across different clinical settings.

Cardiac and thrombotic manifestations were relatively uncommon in this cohort but remain clinically significant. Although observed in a minority of patients, these complications carry substantial prognostic implications in HES and should not be underestimated. Cardiac involvement, in particular, may be asymptomatic in its early stages and can progress to severe complications such as intracardiac thrombosis, endomyocardial fibrosis, valvular disease, and restrictive cardiomyopathy (7, 11, 14). In the present cohort, cardiac involvement was particularly enriched in EGPA, the subtype with the highest organ burden, supporting the biological plausibility of eosinophil-mediated endothelial and myocardial injury. In the baseline work-up, first-level cardiac screening included electrocardiography, cardiac biomarkers (serum assessment of NT-proBNP and Troponin T/I), and transthoracic echocardiography, with advanced cardiac imaging reserved for abnormal or clinically suggestive findings. On this basis, systematic cardiac assessment should be considered, especially in patients with elevated AEC, multiorgan involvement, pulmonary disease, neurological manifestations, or otherwise unexplained systemic symptoms, even in the absence of overt cardiac signs.

Several limitations should be acknowledged. The retrospective, single-center design limits causal inference and may introduce referral bias. The overall sample size and the relatively small number of patients within certain subgroups, particularly myeloid HES, reduce statistical power and limit the robustness of subtype-specific analyses. In addition, some associations rely on sparse events and should be interpreted with caution. The analyses evaluating the relationship between AEC and multiorgan involvement were exploratory and should be interpreted cautiously, as it may be partly influenced by subtype distribution, particularly the high eosinophil counts and multiorgan burden observed in EGPA. Prospective, multicenter studies are needed to validate these findings.

Despite these limitations, this study provides a comprehensive real-world characterization of the relationship between HES subtypes and organ involvement. Organ involvement was common, frequently multiorgan, and unevenly distributed across HES forms. Higher AEC identified patients at increased risk of multiorgan disease but did not replace the need for direct organ assessment. Taken together, these findings support an integrated diagnostic approach that combines etiologic classification with systematic organ phenotyping. Such a strategy may improve diagnostic accuracy, facilitate earlier detection of subclinical organ damage, refine risk stratification, and support more individualized follow-up and therapeutic decision-making in patients with hypereosinophilic disorders.

## Data Availability

The raw data supporting the conclusions of this article will be made available by the authors, without undue reservation.
